# Hepatoprotection and neuroprotection induced by low doses of IGF-II in aging rats

**DOI:** 10.1186/1479-5876-9-103

**Published:** 2011-07-06

**Authors:** Inma Castilla-Cortázar, María García-Fernández, Gloria Delgado, Juan E Puche, Inma Sierra, Rima Barhoum, Salvador González-Barón

**Affiliations:** 1 Department of Medical Physiology, CEU-San Pablo University School of Medicine Institute of Applied Molecular Medicine (IMMA) Boadilla del Monte, 28668 Madrid, Spain; 2 Department of Physiology, School of Medicine, University of Málaga, 29071 Málaga, Spain

## Abstract

**Background:**

GH and IGFs serum levels decline with age. Age-related changes appear to be associated to decreases in these anabolic hormones. We have previously demonstrated that IGF-I replacement therapy improves insulin resistance, lipid metabolism and reduces oxidative damage (in brain and liver) in aging rats. Using the same experimental model, the aim of this work was to study whether the exogenous administration of IGF-II, at low doses, acts analogous to IGF-I in aging rats.

**Methods:**

Three experimental groups were included in this study: young healthy controls (yCO, 17 weeks old); untreated old rats (O, 103 weeks old); and aging rats treated with IGF-II (O+IGF-II, 2 μg * 100 g body weight^-1 ^* day^-1^) for 30 days. Analytical parameters were determined in serum by routine laboratory methods using an autoanalyzer (Cobas Mira; Roche Diagnostic System, Basel, Switzerland). Serum levels of hormones (testosterone, IGF-I and insulin) were assessed by RIA. Serum Total Antioxidant Status was evaluated using a colorimetric assay. Mitochondrial membrane potential was evaluated using rhodamine 123 dye (adding different substrates to determine the different states). ATP synthesis in isolated mitochondria was determined by an enzymatic method.

**Results:**

Compared with young controls, untreated old rats showed a reduction of IGF-I and testosterone levels with a decrease of serum total antioxidant status (TAS). IGF-II therapy improved serum antioxidant capability without modifying testosterone and IGF-I circulating concentrations. In addition, IGF-II treatment reduced oxidative damage in brain and liver, improving antioxidant enzyme activities and mitochondrial function. IGF-II was also able to reduce cholesterol and triglycerides levels increasing free fatty acids concentrations.

**Conclusions:**

We demonstrate that low doses of IGF-II induce hepatoprotective, neuroprotective and metabolic effects, improving mitochondrial function, without affecting testosterone and IGF-I levels.

## Background

The Insulin-like Growth Factors (IGF-I and IGF-II) are ubiquitously expressed growth factors that have profound effects on the growth and differentiation of many cell types and tissues, including cells from the CNS [1-3].

We have previously studied some conditions of "IGF-I deficiency", such as liver cirrhosis and aging, in which replacement therapy could be considered as an effective therapeutic strategy [4-16]. In fact, the exogenous administration of low doses of IGF-I in aging rats was able to reduce oxidative damage (in brain and liver) improving mitochondrial function and antioxidant enzyme activities; and to diminish insulin resistance and improve lipid metabolism [4,5]. These beneficial effects were associated to an increase in testosterone levels.

IGF-II is a peptidic hormone of 67 amino acids that belongs to the family of IGFs. It plays an important role in the embryology development but the physiological functions of IGF-II in the adult life are not fully understood [17,18].

GH, IGF-I and IGF-II concentrations decline with age. Age-related changes appear to be linked to decreases in these anabolic hormones. A significant amount of evidence has been accumulated showing that IGF-I might play a role in several pathological conditions usually observed during aging associated with oxidative damage [3,19-21].

The aim of this work was to investigate whether IGF-II is able to act analogous to IGF-I in aging. Accordingly, this study was designed to examine the effect of low doses of IGF-II in old rats on: anabolic hormones (testosterone, insulin and IGF-I); glucose and lipid metabolisms; total antioxidant status (TAS, as the reductive capability of serum depending on enzyme and non-enzyme molecules) [22]; and on oxidative damage in brain (cortex and hippocampus) and liver.

A similar design was performed [4] using the same markers of lipid peroxidation (Malondialdehyde, MDA) and protein carbonylation (Protein Carbonyl Content, PCC) as well as the parameters of mitochondrial function (mitochondrial membrane potential-MMP- and ATP synthesis). Three experimental groups were included in that protocol: young healthy controls (yCO, 17 weeks old); untreated old rats (O, 103 weeks old); and aging rats treated with IGF-I (O+IGF-I) for 30 days (2.25 μg 100 g body weight^-1 ^day^-1^).

## Methods

### Experimental design

The age of the control animals was selected following the criteria previously described in Garcia-Fernandez M et al. [4]. Thus, 17 weeks old (wk) mice were judged to be a suitable age for young controls (yCO). 103 wk mice were considered suitable to evaluate the decline in anabolic hormones and the reduction in antioxidant capability, as well as to investigate the impact of IGF-II therapy on these parameters in aging animals. The experimental procedure was performed as follow: healthy male Wistar rats were divided into two groups according to age: yCO (n = 8) 17 wk, and aging control rats 103 wk. Old animals were randomly assigned to receive either 0.5 mL saline (group O, n = 8) or 2 μg rhIGF-II 100 g bw^-1^d^-1 ^(Lilly Laboratories, Madrid, Spain) subcutaneously (group O+IGF-II, n = 8) for 30 days. This IGF-II dose was elected by maintaining a similar one used in previous studies with IGF-I (in order to be able to compare their relative actions).

All experimental procedures were performed in compliance with *The Guiding Principles for Research Involving Animals *[23] and approved by the Bioethical Committee from the University of Málaga. Both food (standard semipurified diet for rodents; B.K. Universal, Sant Vicent del Horts, Spain) and water were given *ad libitum*. Rats were housed in cages placed in a room with a 12-h light, 12-h dark cycle and constant humidity and temperature (20°C).

In the morning of the 31st day, blood was obtained from the retroocular plexus with capillary tubes (70 mm; Laboroptik, Marienfeld, Germany) and divided into aliquots that were stored at -20°C until used. The animals were then killed by decapitation. The liver and brain were dissected. Samples from the cortex and hippocampus were stored separately until assaying at -80°C after immersion in liquid Nitrogen.

In six animals from each group, a part of the fresh liver was used to isolate mitochondria and to perform mitochondrial function tests by flow cytometry.

### Analytical methods in serum

Analytical parameters (total protein, glucose, cholesterol, triglycerides, free fatty acids, aspartate transaminase and alanine transaminase) were determined in serum by routine laboratory methods using an autoanalyzer (Cobas Mira; Roche Diagnostic System, Basel, Switzerland).

Serum levels of hormones (testosterone, IGF-I and insulin) were assessed by RIA in a GammaChen 9612 Plus (Serono Diagnostics, Roma, Italy) using specific commercial assay systems: a) free testosterone by a Coat-a-Count, DPC (Diagnostic Products Corp., Los Angeles, CA). The sensitivity of total testosterone assay was 4 ng/dL, and the intraassay coefficient of variation was less than 7%; b) assessment of serum IGF-I levels were performed using radioimmunoassay with coated tubes for the determination of IGF-I (IGF binding protein blocked), which included elimination of interference by IGF binding protein through excess IGF-I as well as acid alcohol (ALPCO Diagnostics, Windham, NH). The sensitivity of IGF-I assay was 0.1 ng/mL and the interserial coefficient of variation was 7.4; c) Insulin levels were determined using a specific kit for RIA (LINCO Research, Inc., St. Charles, MO) following protocol instructions. The sensitivity was 0.1 ng/mL.

The homeostasis model assessment (HOMA), as an index of insulin resistance, was assessed using the HOMA Calculator Program version 2.0 based on the HOMA formula [24,25]. The HOMA estimates steady state β-cell function (percent B) and insulin S (percent S) as percentages of a normal reference population. These measures correspond well but are not necessarily equivalent to non steady state estimates of β-cell function and insulin S derived from stimulatory models such as the hyperinsulinemic clamp, hyperglycemic clamp, iv glucose tolerance test (acute insulin response, minimal model), and the oral glucose tolerance test (0-30 δ insulin/glucose ratio).

Serum Total Antioxidant Status [26], as total enzymatic and non enzymatic antioxidant capability of serum, was evaluated using a colorimetric assay (Randox Laboratories Ltd., CrumLin, UK) using the following principle: Abts [2,2´-Azino-di-('3-ethylbenzthiazoline sulfonate)] was incubated with a peroxidase (metmyoglobin) and H_2_O_2 _to produce the radical cation Abts^+^. This has a relatively stable blue-green color, which is measured at 600 nm. Antioxidants in the added sample cause suppression of this color production to a degree that is proportional to their concentration [22].

### Parameters of oxidative damage and antioxidant defenses in liver and brain homogenates

#### Lipid peroxidation

Malondianldehyde (MDA) was used as an index of lipid peroxidation [27] in liver and brain homogenates, and it was measured after heating samples at 45°C for 60 min. in acid medium. It was quantified by a colorimetric assay using LPO-586 (Bioxytech; OXIS International Inc., Portland, OR), which after reacting with MDA generates a stable chromophore that can be measured at 586 nm (Hitachi U2000 Spectro; Roche Molecular Biochemicals, Basel, Switzerland) [26,27].

#### Protein Carbonyl Content (PCC)

PCC was determined by the method of Levine *et al*. [28]. Each sample was divided into two portions containing 1 to 2 mg protein each. An equal volume of 2 M HCl was added to one portion, incubated at room temperature for 1 h, and shaken intermittently. The other portion was treated with an equal volume of 10 mM dinitrophenylhydrazine in 2 M HCl and incubated for 1 h. at room temperature. After incubation, the mixture was precipitated with 10% trichloroacetic acid and centrifuged.

The precipitate was washed with ethanol-ethyl acetate (1:1), twice dissolved in 1 mL 6 M guanidine HCl, centrifuged at low speed, and the supernatant was extracted. The difference in absorbance between the dinitrophenylhydrazine-treated and HCl-treated samples was determined at 366 nm.

#### Antioxidant Enzyme Activities

Superoxide dismutase (SOD) [Enzyme Commission of the International Union of Biochemistry (EC) 1. 15. 1. 1.], catalase (EC 1. 11. 1. 6.) and glutathione peroxidase (GPX: EC 1. 11. 1. 9.) activities were measured both in brain and liver tissues. Samples were homogenized in Tris-HCl buffer [20 mmol/L (pH 7.4); 1 g tissue/10 mL] at 0°C and centrifuged at 25,000 g for 30 min at 4°C. All measurements were performed in the supernatant. SOD activity was determined at 37°C using a commercial kit (Randox Laboratories) and an autoanalyzer (Cobas Mira; Roche Diagnostic System, Basel, Switzerland). Catalase activity was determined at 25°C by measuring the changes of absorbance using final concentrations of 10 mmol/L H_2_O_2 _and 50 mmol/L phosphate buffer (pH 7.0) at 240 nm during the time interval 15-30 sec after addition of the sample [26,29,30]. GPX was measured at 37°C with the Ransod commercial kit using the Cobas Mira autoanalyzer, and its activity was expressed in units (1 U equals to 1 μmol substrate turnover/min) per milligram of protein [30].

GRD (EC 1. 6. 4. 2.) was measured in brain homogenates. Antioxidant activity was determined at 37°C using a commercial kit (Ransod; Randox Laboratories) and the same autoanalyzer as above.

### Isolation of liver mitochondria

Liver mitochondrial fraction was prepared according to the method described by Hogeboom and Schneider [31] with modifications. Liver samples were homogenized (1:10 wt/vol) in an ice-cold isolation buffer (pH 7.4) containing sucrose 0.25 M, KH_2_PO_4 _5 mM, 3[N-morholino]propanesulfonic acid 5 mM, and 0.1% BSA. The homogenate was centrifuged at 800 × *g *for 10 min. The resulting supernatant was centrifuged at 8,500 × *g *for 10 min. The supernatant was discarded, and the pellet diluted in cold isolation buffer and centrifuged at 8,500 × *g *for 10 min. three times. The final mitochondria pellet was resuspended in a minimal volume, and aliquots were stored at -80°C until used in enzyme assays. All procedures were conducted at 4°C.

### Flow cytometry analysis in isolated mitochondria

#### Mitochondrial Membrane Potential (MMP)

MMP was evaluated using rhodamine 123 dye (RH123) obtained from Molecular Probes, Inc. (Eugene, OR). It has an absorbance maximum of 500 nm and an emission maximum of 523 nm. Mitochondrial suspensions (40 to 50 μg protein mitochondria/mL) were incubated in isolation buffer for 5 min. in the dark with RH123 (0.5 μg/mL), after adding various agents. For state 4 conditions, the mitochondrial samples were incubated with rotenone 25 μM and energized with sodium succinate 5 mM, and with rotenone 25 μM, sodium succinate 5 mM plus ADP 200 μM for state 3 conditions. The uncoupler valinomycin 10 μM was added to confirm that the uptake of RH123 was related to MMP [32]. Finally, 2.5 μg/mL oligomycin (Sigma-Aldrich, St. Louis, MO) was added as an inhibitor of ATP synthetase to block all phosphorylation-related respiration [32].

### ATP synthesis by mitochondria

ATP synthesis was assessed [33] by resuspending a mitochondrial population in buffer and supplemented with 5 mM KH_2_PO_4_, 5 mM succinate, and 5 mM ADP, and then incubated for 15 min at 37°C. The reaction was stopped by adding an equal volume of 12% trichloroacetic acid, and the reaction mixture was centrifuged at 1,200 g for 15 min. at 4°C. The supernatant was neutralized with 6 N KOH and measured for ATP by an enzymatic method using a kit obtained from Sigma Chemical (Tokyo, Japan).

### Statistical analysis

Data are expressed as mean ± SEM. To assess the homogeneity among the different groups of rats, a Kruskal-Wallis test was used, followed by multiple *post hoc *comparisons using Mann-Whitney U tests. A regression model was fitted considering SOD and MDA as the independent and dependent variables, respectively. Any P value less than 0.05 was considered statistically significant.

Calculations were performed with SPSS in v.15.0 program (SPSS, Inc., Chicago, IL).

## Results

The experimental model of aging was previously characterized [4] attending to the evolution of serum levels of IGF-I, free testosterone and total antioxidant status.

### Effect of IGF-II therapy on anabolic hormones and serum total antioxidant status

Consistent with previous studies [4], old animals showed a reduction in IGF-I and testosterone levels and a statistically significant increase of insulin levels (see Table 1). In contrast with IGF-I therapy, IGF-II treatment was not able to increase testosterone circulating levels and slightly reduced insulin levels. No changes were observed in IGF-I circulating concentrations (see Table 1).

**Table 1:** Circulating levels of testosterone, IGF-I and insulin in the three experimental groups.

	Young controls (yCO) (n = 8)	Untreated old rats (O) (n = 8)	Old rats treated with IGF-II (O+IGF-II) (n = 8)
Free testosterone (pg/mL)	6.50 ± 1.16	4.01 ± 0.76^a^	4.07 ± 1.34^a^
IGF-I (ng/mL)	978.92 ± 63.66	629.12 ± 16.56^a^	656.00 ± 25.57^a^
Insulin (ng/mL)	0.45 ± 0.08	1.54 ± 0.43^a^	1.12 ± 0.10
Total Antioxidant Status (mmol/L)	0.87 ± 0.01	0.79 ± 0.01^a^	0.87 ± 0.02^b^

Effect of IGF-II treatment on serum total antioxidant status.

Values are expressed as ± SEM.

^a ^*P *< 0.05 vs yCO

^b ^*P *< 0.05 vs O

However, IGF-II treatment significantly increased serum total antioxidant capability (TAS), reaching similar values to those reported with the IGF-I replacement therapy [4].

### Effect of IGF-II therapy on glucose and lipid metabolisms

**Figure 1:**
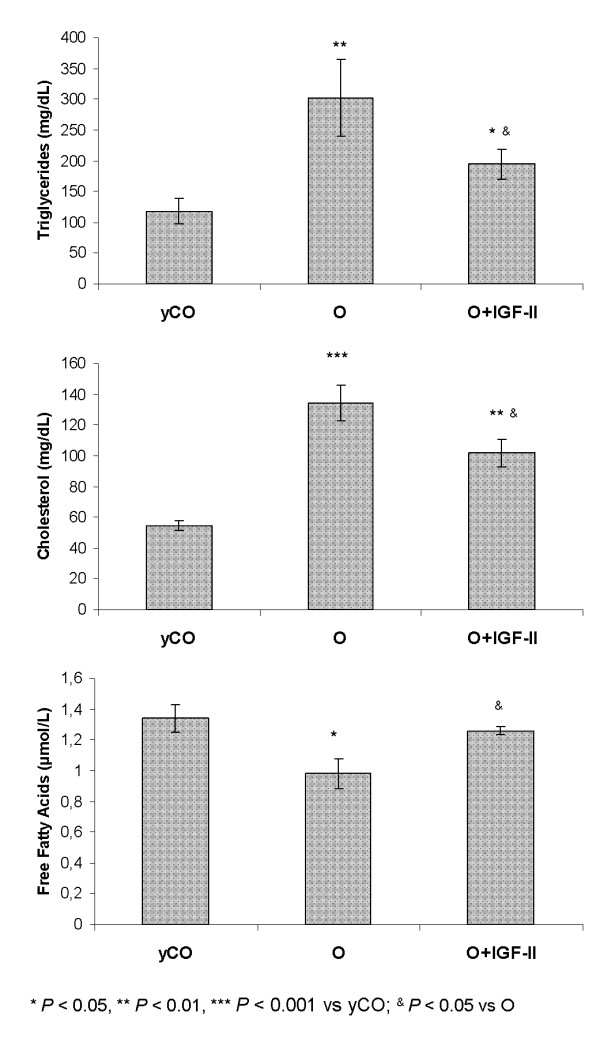
**Effect of IGF-II therapy on lipid metabolism**.

Of interest, untreated aging rats (O group) showed an increase of cholesterol and triglycerides circulating levels that IGF-II therapy reduced by increasing free fatty acids (Figure 1). These effects on lipid metabolism were similar to those found previously with IGF-I replacement therapy [4].

Table 2 summarizes the analytical parameters in the three experimental groups. IGF-II therapy was not able to reduce neither blood glucose levels (in contrast with IGF-I treatment [4]) nor total protein (as previously shown by IGF-I treatment [4]). However, it was only found a slight effect on insulin resistance (HOMA index).

**Table 2:** Analytical parameters in the three experimental groups

	Young controls (yCO) (n = 8)	Untreated old rats (O) (n = 8)	Old rats treated with IGF-II (O+IGF-II) (n = 8)
Total Proteins (g/L)	56.33 ± 0.47	63.08 ± 1.23^a^	67.83 ± 0.48^a^
Glucose (mmol/L)	4.10 ± 0.41	5.49 ± 0.22^b^	5.44 ± 0.19^b^
HOMA	1.17 ± 0.45	5.95 ± 1.55^c^	3.05 ± 0.56^bd^
ALT (U/L)	25.93 ± 1.14	26.41 ± 2.47	30.33 ± 2.61
AST (U/L)	99.84 ± 5.60	86.26 ± 5.41	108.33 ± 5.91

Values are expressed as ± SEM. ALT, Alanine transaminase; AST, aspartate transaminase

^a ^*P *< 0.001 vs yCO

^b ^*P *< 0.05 vs yCO

^c ^*P *< 0.01 vs yCO

^d ^*P *< 0.05 vs O

### Oxidative damage and antioxidant enzyme activities in brain and liver, in the three experimental groups

**Figure 2:**
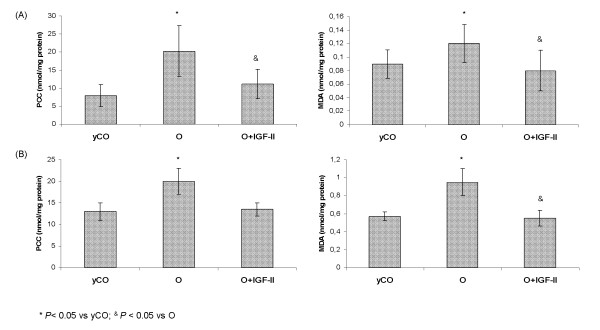
**Parameters of oxidative damage in brain: protein carbonylation (PCC) and lipid peroxidation (MDA)**. (A) In cortex; (B) In hippocampus.

Parameters of oxidative damage in brain (cortex and hippocampus) in the three experimental groups are shown in Figure 2. Untreated old rats (O) presented significant increases of lipid peroxidation products (MDA, expressed as nmol/mg protein; and PCC, expressed as nmol/mg protein) compared with yCO (cortex MDA: O = 0.12 ± 0.02 *vs*. yCO = 0.09 ± 0.01, *P *< 0.05; cortex PCC: O = 20.21 ± 3.91 *vs*. yCO = 7.90 ± 1.19, *P *< 0.05; hippocampus MDA: O = 0.95 ± 0.15 *vs*. yCO = 0.57 ± 0.05, *P *< 0.05; and hippocampus PCC: O = 20.35 ± 2.94 *vs*. yCO = 13.11 ± 2.07, *P *< 0.05).

Low doses of IGF-II were able to reduce significantly both parameters of oxidative damage (cortex MDA: O+IGF-II = 0.08 ± 0.02; cortex PCC: O+IGF-II = 11.13 ± 4.10; hippocampus MDA: O+IGF-II = 0.55 ± 0.09; and hippocampus PCC: O+IGF-II = 13.56 ± 1.54, nmol/mg protein), reaching values similar to those found in young controls (*P = *ns).

**Figure 3:**
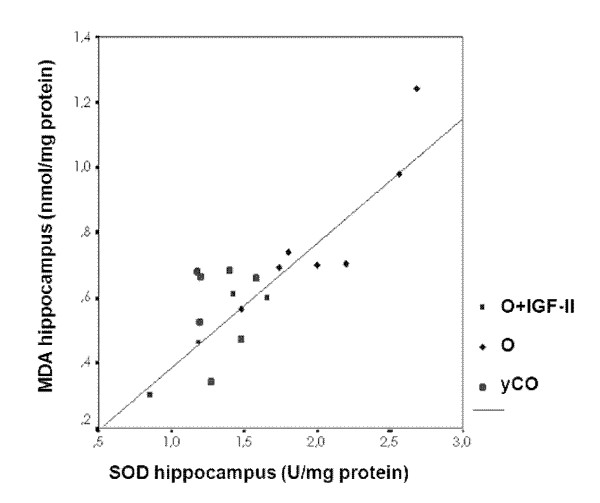
**Direct and significant correlation between the specific activity of SOD and the marker of lipid peroxidation MDA in hippocampus**.

Regarding antioxidant enzyme activities, several statistically significant alterations were found in untreated aging rats (O group) compared with yCO, which are summarized in Table 3. Old rats treated with IGF-II showed similar values to those found in yCO (*P = *ns yCO *vs*. O+IGF) with the only exception of GPX in hippocampus (*P *< 0.05). A close correlation was found between SOD activity and MDA in hippocampus: Figure 3.

**Table 3:** Antioxidant enzymes activities in brain and liver

		Young controls (yCO) (n = 8)	Untreated old rats (O) (n = 8)	Old rats treated with IGF-II (O+IGF-II) (n = 8)
In cortex	**Catalase **(U/mg prot)	22.8 ± 0.85	18.90 ± 1.05^a^	20.2 ± 1.2^b^
	**SOD **(U/mg prot)	3.99 ± 0.09	5.32 ± 0.38^a^	4.8 ± 0.2
	**GPX **(mU/mg prot)	89.3 ± 2.85	77.9 ± 2.85^a^	108 ± 5^b^
	**GRD **(U/mg prot)	41.80 ± 0.09	42.75 ± 1.9	46 ± 2

In hippocampus	**Catalase **(U/mg prot)	9.40 ± 0.47	15.01 ± 2.09^a^	7.9 ± 1.1^c^
	**SOD **(U/mg prot)	1.23 ± 0.09	1.99 ± 0.19^d^	1.3 ± 0.2^c^
	**GPX **(mU/mg prot)	55.1 ± 2.85	83.6 ± 4.75^d^	66 ± 5
	**GRD **(U/mg prot)	31.35 ± 0.09	42.75 ± 2.85^a^	35 ± 4

In liver	**Catalase **(KU/mg)	2.85 ± 0.28	1.90 ± 0.19^b^	2.70 ± 0.60
	**SOD **(U/mg)	11.59 ± 1.71	12.25 ± 1.99	18.20 ± 2.00
	**GPX **(U/mg)	1.04 ± 0.09	2.09 ± 0.19^b^	2.10 ± 0.10^b^

Values are expressed as ± SEM. prot, Protein.

^a ^*P *< 0.01 vs yCO

^b ^*P *< 0.05 vs yCO

^c ^*P *< 0.05 vs O

^d ^*P *< 0.001 vs yCO

In liver homogenates, MDA was significantly increased in untreated old rats (O = 0.19 ± 0.02 *vs*. yCO = 0.10 ± 0.01; *P *< 0.001), and IGF-II therapy also reduced this marker of lipid peroxidation (O+IGF-II = 0.16 ± 0.01, *P *< 0.01 vs yCO and *P *< 0.05 vs O). Not statistically significant differences were found in PCC between the three experimental groups (yCO = 3.41 ± 0.82; O = 4.21 ± 1.38; and O+IGF-II = 3.88 ± 1.68 nmol/mg protein; *P *= ns) in liver. Results regarding antioxidant enzymes activities in liver are summarized in Table 3. Only significant differences between groups were found in catalase, which was decreased in untreated old rats (*P *< 0.05), whereas IGF-II-treated old rats presented similar values to those found in yCO. All these data are similar to those found with IGF-I therapy in aging rats [4].

### Mitochondrial Membrane Potential (MMP) and ATP synthesis in liver mitochondria

**Figure 4:**
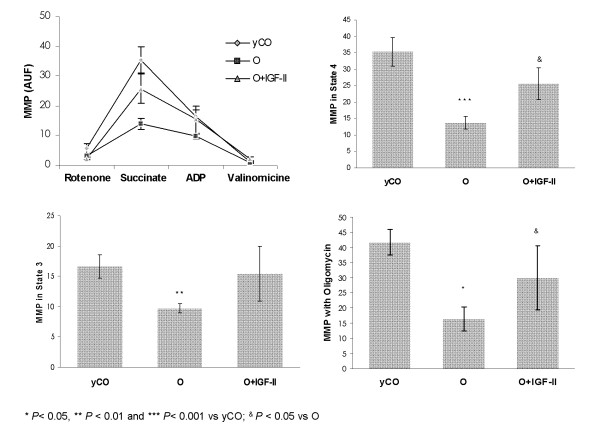
**Mitochondrial Membrane Potential (MMP) in isolated liver mitochondria by flow cytometry**. MMP is considered a good marker of mitochondrial function [32] which is assessed under different substrates. Mitochondria from untreated aging rats (O group) showed a significant depletion of MMP in all conditions. AUF, arbitrary units of fluorescence.

Figure 4 summarizes the MMP with different substrates, which is considered a good marker of mitochondrial function. A reduction of MMP was observed in untreated aging rats in all conditions compared with yCO group: state 4 (*P *< 0.001 *vs*. yCO), state 3 (*P *< 0.01 *vs*. yCO), and with oligomycin (*P *< 0.05 *vs*. yCO), whose deactivating ATPase shows the condition of maximum intramitochondrial negativity.

Mitochondria from old rats treated with IGF-II therapy presented similar values to those found in mitochondria from yCO group (*P = *ns) in all conditions (state 4: yCO = 34.23 ± 4.05, O = 13.25 ± 2.15, *P *< 0.001; O+IGF-II = 25.67 ± 4.34 *P *< 0.05 vs O) (state 3: yCO = 15.95 ± 2.50, O = 8.80 ± 1.10, *P *< 0.05; O+IGF-II = 15.40 ± 3.60, P = ns vs yCO) (with oligomycin: yCO = 41.75 ± 4.10, O = 16.35 ± 4.10, *P *< 0.05; O+IGF-II = 29.89 ± 6.25, *P *< 0.05 vs O)

**Figure 5:**
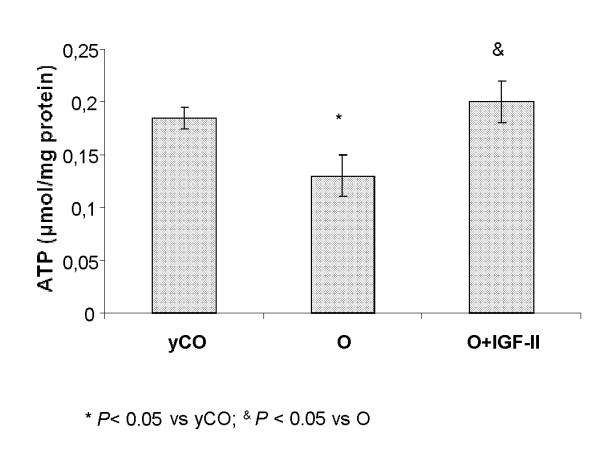
**ATP synthesis in isolated mitochondria from the three experimental groups**: IGF-II therapy increased ATP production.

Accordingly, ATP production was reduced in untreated aging rats (yCO = 0.18 ± 0.01 *vs*. O = 0.13 ± 0.01 μmol/mg mitochondrial protein), and IGF-II therapy was able to recover ATP production, reaching similar values to those found in yCO (O+IGF-II = 0.20 ± 0.02 μmol/mg mitochondrial protein): Figure 5.

## Discussion

GH/IGFs axis declines with age. We have previously considered that aging is a novel "condition of IGF-I deficiency" since circulating levels of this hormone are reduced, anabolism is diminished and oxidative stress is one of the most important mechanisms of cellular damage in aging [4,26]. IGF-II levels are also reduced in aging [34]. IGF-II is expressed in the brain and surrounding structures [1]. However, the physiological role of IGF-II in adult life is not fully understood.

Although there are interesting discrepancies for understanding the physiological relevance of the reduced concentrations of GH and IGFs in aging, it is well established that IGF-I replacement therapy induces beneficial effects including neuroprotection and hepatoprotection as well as a reduction of insulin resistance and lipid circulating levels increasing testosterone [4,5].

In the present study, we analyzed whether low doses of IGF-II are able to induce similar effects that those reported with IGF-I [4,5] acting analogous to IGF-I in aging rats.

Consistent with previous findings, old rats showed hyperlipidemia and insulin resistance, increased brain and hepatic oxidative damage and reduced testosterone circulating levels, compared with young controls. In addition, untreated aging rats showed a mitochondrial dysfunction with depletion of MMP and a significant reduction of ATP synthesis.

The major finding in this study is the recognition that the exogenous administration of low doses of IGF-II is able to exert beneficial effects on age related changes without increasing testosterone levels. This occurred through reducing oxidative damage on brain and liver as shown by normalization of mitochondrial dysfunction and antioxidant enzyme activities. Therefore, these results allow us to clearly discerning that the outlined cytoprotective effects are owed to specific actions of the IGFs.

In contrast with the action of IGF-I replacement therapy [4], in this study IGF-II therapy reduced brain oxidative damage without increasing testosterone levels. It has been suggested that the decrease in some hormones in aging, such as estradiol and IGF-I, may have a negative impact on brain function since estradiol and IGF-I signaling interact to promote neuroprotection [35].

Of interest, results in this paper show that IGF-II at low doses induces neuroprotection reducing lipid peroxidation and protein carbonylation irrespective of testosterone or IGF-I circulating levels.

Mountain of recent evidences indicate the role of IGFs on neuronal plasticity, neurogenesis, dendritic branching and synaptogenesis [3,21,36]. Indeed, IGF-I and IGF-II have been suggested as potent neuronal mitogens and survival factors [37]. And interestingly, it has also been recently reported that IGF-II administration in rats significantly enhances memory retention and prevents forgetting [38], suggesting this hormone as a possible novel target for cognitive enhancement therapies.

Oxidative damage is considered a key mechanism of cellular damage in aging and the ensuing oxidative stress leads to lipid peroxidation, mitochondrial dysfunction and ATP depletion [26,39,40]. In turn, injured mitochondria and products of lipid peroxidation further induce cell damage [39,40]. Results in this paper suggest that the observed cytoprotective actions of IGF-II may be due to decreased peroxidative cell damage. In an attempt to characterize the protection afforded by IGF-II against free radical damage, we investigated the effect of IGF-II therapy on antioxidant enzymes and mitochondrial function following the same protocol used to study the effects of IGF-I [4]. Low doses of IGF-II were able to induce the same cytoprotective actions that IGF-I in aging, by reducing the oxidative damage in brain and liver as a result of improved antioxidant enzyme activities and mitochondrial function increasing ATP production. However, IGF-II therapy did not show any relevant effect on glucose metabolism. Whereas IGF-I replacement therapy significantly reduced hyperglucemia and hyperinsulinemia in old rats diminishing insulin resistance index (HOMA) [4], non-relevant effects were induced by IGF-II since hyperglucemia was not reduced.

Another point that deserves particular mention is that IGF-II treatment significantly improved lipid metabolism, as shown by reduced cholesterol and triglycerides and increased free fatty acid levels.

## Conclusions

This study demonstrates that exogenous administration of low doses of IGF-II exerts beneficial effects on age-related changes in rats without increasing their testosterone levels. The mechanism involves reduced oxidative damage on brain and liver as shown by normalization of mitochondrial dysfunction and antioxidant enzyme activities. Therefore, cytoprotective effects of low doses of IGF-II reported in this work suggest that a therapeutic approach targeted at lowering oxidative damage and improving lipid metabolism could be effective in aging. IGF-II may be considered, in part, as an analogue of IGF-I that does not increase testosterone levels. This fact might be an advantage for elderly people with a testosterone-dependent disease.

## List of Abbreviations

AUF: arbitrary units of fluorescence; bw: body weight; CNS: Central Nervous System; EC: Enzyme Commission of the International Union of Biochemistry; GSHPx: glutathione peroxidase; HOMA: homeostasis model assessment; IGF: Insulin-Like Growth Factor; MDA: malondialdehyde; MMP: mitochondrial membrane potential; ns: not significant; O: untreated old rats; O+IGF-II: aging rats treated with IGF-II; PCC: protein carbonyl content; RH123: rhodamine 123 dye; S: sensitivity; SOD: superoxide dismutase; TAS: total antioxidant status; Yco: young controls.

## Competing interests

The authors declare that they have no competing interests. These results have been registered as P200601523.

## Authors' contributions

ICC designed the research, carried out the in vivo protocol and wrote the paper; MGF, GD, JEP, and IS performed the research; RB analyzed the data; and SGB revised the manuscript.

All authors have read and approved the final manuscript.
